# First evidence of sensory atypicality in mothers of children with Autism Spectrum Disorder (ASD)

**DOI:** 10.1186/2040-2392-5-26

**Published:** 2014-04-03

**Authors:** Mirko Uljarević, Margot R Prior, Susan R Leekam

**Affiliations:** 1 Wales Autism Research Centre, School of Psychology, Cardiff University, 70 Park Place, Cardiff, Wales CF10 3A, UK; 2 Melbourne School of Psychological Sciences, University of Melbourne, Melbourne, VIC 3010, Australia

**Keywords:** Sensory atypicality, Parents, Autism spectrum disorders

## Abstract

**Background:**

Atypical reactions to sensory stimuli show heritability in the general population and are a known risk factor for affective disorders. As sensory problems are highly prevalent in individuals with ASD and their siblings, and the occurrence of affective disorders is elevated in parents of children with ASD, investigating sensory symptoms in parents is important both from clinical and theoretical standpoints.

Fifty mothers of children and adolescents with ASD completed the Adolescent and Adult Sensory Profile (AASP). The AASP is a norm-referenced questionnaire that provides scores for four types of responses to sensory stimuli (sensory quadrants): hypo-sensitivity, hyper-sensitivity, sensation seeking, and sensory avoiding.

**Findings:**

Mothers’ scores were compared with AASP norms. Ninety eight percent of mothers had sensory scores at least one standard deviation (SD) above the normative mean and 44% were two or more SDs above the mean for at least one sensory quadrant.

**Conclusions:**

This study provides the first evidence for sensory atypicality in parents of children with ASD. Further research is needed to elucidate the contribution of genetic and environmental influences on the expression of sensory problems in ASD.

## Introduction

Sensory systems provide pathways for the brain to receive, organize, and make sense of information about the world. These processes are foundational for learning and are necessary for enabling adaptive responses to the environment [1]. Atypical reactions to sensory stimuli are found in individuals with neurodevelopmental and psychiatric conditions including anxiety, Attention Deficit Hyperactivity Disorder (ADHD), Fragile X syndrome and schizophrenia [2]. Sensory atypicalities are particularly common in individuals with Autism Spectrum Disorders (ASD), with the majority of studies reporting prevalence of above 90% for both children and adults [3]. Sensory atypicalities have become increasingly recognised within the diagnostic criteria for ASD with ‘hyperreactivity or hyporeactivity to sensory input, or unusual interests in sensory aspects of environment’ now explicitly included as a symptom subdomain in the latest version of the Diagnostic and Statistical Manual of Mental Disorders (DSM) criteria for ASD (5^th^ edition; DSM-5; [4]).

There is evidence for a genetic influence on sensory atypicalities [5,6]. Goldsmith *et al*. [7] estimated twin similarity on a perceptual sensitivity scale from the Children’s Behavior Questionnaire finding that monozygotic and dizygotic twin correlations were .58 and .37, respectively. Given that ASD itself is a highly heritable disorder [8] with subclinical autistic traits found in parents [9], it is surprising that the presence of these problems has not been studied in parents of children with ASD.

Increased understanding of sensory atypicality in parents of children with ASD may contribute to knowledge about their clinical profile. Affective disorders are more frequent in parents of children with ASD than in parents of children with other developmental conditions [10]. Although the reasons for such a high prevalence of affective disorders are poorly understood, there is evidence that sensory atypicalities present a risk for affective disorders in the general population [11]. Therefore, the aim of this study was to assess the presence of sensory atypicalities in parents of children and adolescents with ASD.

## Methods

### Participants

Fifty mothers of children and adolescents with ASD (mean age of children = 10 years 7 months (10.7), standard deviation (SD) = 3.10; mean age of mothers = 44.4, SD = 6.3) living in South Wales, UK, were recruited to the study. All children had a community multidisciplinary team assessment leading to a best estimate clinical diagnosis of an ASD according to DSM-IV-TR [12] and International Classification of Diseases, version 10 (ICD-10) [13] criteria. In addition, data from the Social Communication Scale [SCQ; [14] were available for all children in the appropriate developmental range (N = 45) and all scored above 15 with the exception of two who scored 14. Data reported here were part of a larger study in which a set of questionnaires was sent to each family for completion by either parent. In all cases it was mothers who responded. None of the mothers had a diagnosis of ASD. Socioeconomic status (SES) data were not available but data on educational level showed that 32% of mothers had postgraduate qualifications, 30% had undergraduate or vocational qualifications and 25% did not have post-school qualifications (12.3% declined to give education information).

### Procedures and measures

The study was approved by the Cardiff University School of Psychology Research Ethics Committee. Parents were recruited through local schools and parent support groups. Most mothers completed and returned the questionnaire by post. A small proportion (16%) chose to complete the questionnaire as part of the visit to the university.

The Adolescent/Adult Sensory Profile (AASP) [15]. is a 60-item self-report questionnaire containing statements about different responses to various sensory stimuli. Individuals rate the frequency of behaviour described in each statement on a five-point Likert Scale (score of five indicates higher endorsement of the item (that is, more atypicality). This is the opposite of the rating system on the Sensory Profile where a lower score indicates more atypicality). Sensory features are measured using Dunn’s model [16] that classifies individuals on two dimensions: their neurological threshold (high/low) and behavioural response (active/passive). Based on the interaction between these dimensions, patterns of sensory processing are classified into four quadrants. A high threshold combined with a passive response is described as low registration (example item: ‘I don’t seem to notice when someone touches my arm or back’); a high threshold with an active response is described as sensation seeking (‘I like to go to places that have bright lights and that are colourful’); a low threshold combined with passive response is described as sensory sensitivity (‘I become bothered when I see lots of movement around me’); and a low threshold with an active response is described as sensation avoiding (‘I stay away from noisy settings’) quadrant. These quadrants reflect an individual’s pattern of responding across modalities. AASP is a norm referenced questionnaire with cut off scores from a large normative sample. Each quadrant consists of 15 items. Based on those scores, an individual’s performance on each of the quadrants can be classified in the five following categories: (1) Much Less Than Other People, (2) Less Than Other People, (3) Similar to Other People (Typical Performance), (4) More than Most People, or (5) Much More than Most People. It is possible for a single individual to have atypical scores in more than one sensory quadrant.

## Findings

Descriptive statistics and internal consistency of AASP quadrants are presented in Table 1 together with mean raw scores for the AASP normative sample. Initial data screening revealed no outliers and no missing data.

**Table 1:** Descriptive statistics

**Variables**	**Mothers of children with ASD**			**Normative sample**	
	**Mean (SD)**	**Range**	**Cronbach’s alpha**	**Mean (SD)**	**Cronbach’s alpha**
Low registration	38.84 (10.24)	19 to 58	.796	30.29 (6.25)	.82
Sensory seeking	40.32 (8.65)	24 to 67	.701	49.91 (6.83)	.79
Sensory sensitivity	41.60 (11.70)	15 to 65	.795	33.71 (7.63)	.81
Sensory avoidance	41.35 (12.08)	19 to 63	.866	34.57 (7.34)	.66

In the original normative sample [15], 68% showed typical performance, with 28% having scores between one and two SDs outside of normative range and between 2% and 4% with scores of two or more SDs outside of the normative range. In contrast, 98% of mothers of children with ASD scored at least one SD above or below the normative mean for at least one sensory quadrant (32% for one, 18% for two and 48% for either three or four sensory quadrants). Moreover, 44% scored two or more SDs outside the normal range for at least one sensory quadrant (20% for one, 8% for two and 16% for three sensory quadrants). Table 2 provides the classification distributions of mothers for each of the four quadrants. In comparison with typically developing (TD) norms, 62% scored higher on sensory hypo-sensitivity (also known as the low registration) quadrant, 44% higher for the sensory sensitivity quadrant, 48% higher for the sensory avoidance quadrant and 60% of mothers had lower sensory seeking scores than the TD norms.

**Table 2:** Performance of parents across four sensory quadrants

**Quadrants**	**Response classification**				
	**Two or more SDs below the mean**	**Between 1 and 2 SDs below the mean**	**Typical performance (less than 1 SD above or below the mean)**	**Between 1 and 2 SDs above the mean**	**Two or more SDs above the mean**
Low registration	0	1 (2%)	18 (36%)	17 (34%)	14 (28%)
Sensation seeking	15 (30%)	15 (30%)	18 (36%)	1 (2%)	1 (2%)
Sensory sensitivity	2 (4%)	1 (2%)	25(50%)	7 (14%)	15 (30%)
Sensation avoiding	0	7 (14%)	19 (38%)	11 (22%)	13 (26%)

## Discussion

This is the first study to provide evidence of sensory atypicality in parents of individuals with ASD. An exceptionally high number of mothers in this study (49 of 50 (98%) had AASP scores that were atypical; almost half of the sample (22 mothers) scored at least two SDs outside the normative range (only between 2% and 4% of the normative sample had scores in this range).

To date, only one study has looked at the presence of sensory atypicalities in non-twin siblings of ASD individuals who themselves do not have ASD [17]. The AASP was used to examine sensory processing in 80 ASD adolescents, their 56 non-affected adolescent siblings, and 33 adolescent controls. Results showed that compared with typical controls, non-affected autism siblings exhibited significantly fewer sensory seeking behaviours. The authors suggested, therefore, that sensory atypicalities might be a candidate endophenotype, since they meet some of the criteria proposed by Bearden and Freimer [18], namely, that the trait should co-occur with the condition of interest, should co-segregate with the disorder in families, and non-affected family members should express the trait more than the general population.

Since evidence of a positive correlation between the presence of sensory atypicalities and autistic traits in the general population has already been reported [19], it might be argued that our findings can be simply explained by ASD traits-sensory atypicalities association. However, none of the mothers in the study had an ASD diagnosis and we believe that a more complex explanation is likely. Sensory atypicalities are by no means specific to the ASD population and levels of atypicality beyond the general population have been reported in individuals with anxiety and depression, schizophrenia, ADHD and other neuropsychiatric and neurodevelopmental conditions [2]. This raises the question of specificity of the ASD traits-sensory atypicalities relationship. To understand further the role of sensory atypicalities, a large-scale systematic study examining the relationship between sensory atypicality, other ASD traits and co-morbid conditions in family members is needed. Sensory atypicalities are associated with affective disorders in both general [11] and clinical populations including ADHD and ASD [20,21]. Therefore, their contribution to anxiety in parents of children with ASD deserves further attention. The contribution may be direct as inability to tolerate sensory stimuli may impact directly on parental stress and anxiety. It may also be indirect as sensory problems can affect the type of coping strategies that individuals adopt in particular situations [22]. There is evidence that parents of children with ASD, when compared to parents of typically developing children and parents of children with other neuro-developmental disorders, use more avoidant coping strategies and that a higher use of escape-avoidance is associated with higher levels of anxiety [23]. Future research should examine how sensory atypicalities in parents of children with ASD contribute to their level of anxiety and use of coping strategies, preferably using a longitudinal design.

This study had a number of limitations. Results are limited by the sample size and by the use of a self-report questionnaire. We were also able to only recruit mothers of children with ASD. Difficulties related to engaging fathers in research of this kind are not specific to this study and are also found in research on parents of ASD children, typically developing children and children with other neurodevelopmental and psychiatric conditions [24]. Inclusion of fathers in future research is important as research with typically developing children [25] and ASD adults [26] indicates that sensory problems are more prevalent in females and it would be important to explore whether the same trend is present in parents of children with ASD. Future work should include replication of these results with a larger sample and the use of carefully designed experimental protocols to disentangle the neurophysiological mechanisms underlying sensory atypicalities. Furthermore, comparing sensory atypicalities in parents of children with ASD with parents of children with other neurodevelopmental conditions is important. Finally, this study did not include a control group. As this was the pilot study and the first time that sensory atypicalities were assessed in parents of children with ASD, norms from the AASP were considered a good comparison as these are based on a large, representative sample of individuals without any co-morbid conditions in the appropriate age range. However, in future it will be important to compare sensory problems in parents of children with ASD with parents of children with other neurodevelopmental conditions.

## Conclusions

This study is the first to demonstrate that sensory atypicalities are prevalent in mothers of children and adolescents with ASD.

## Abbreviations

AASP: Adolescent and Adult Sensory Profile; ADHD: Attention Deficit Hyperactivity Disorder; ASD: Autism Spectrum Disorder; DSM: Diagnostic and Statistical Manual of Mental Disorders; ICD: International Classification of Diseases; SCQ: Social Communication Questionnaire; SD: standard deviation; SES: socioeconomic status; TD: typically developing.

## Competing interests

The authors declare that they have no competing interests.

## Authors’ contributions

MU participated in the conception, design, data collection and analysis, draft, revision and final approval of the manuscript. SL contributed to the design, draft, revision and final approval of the manuscript, and financial support for dissemination. MP participated in the revision and final approval of the manuscript. All authors read and approved the final manuscript.
